# An Empirical Analysis of the Determinants of Mortality Risks of Chinese Centenarians in the Era of Longevity

**DOI:** 10.1093/geroni/igaa057.1091

**Published:** 2020-12-16

**Authors:** Jiehua Lu, Hui Zhu

**Affiliations:** 1 Peking University, Beijing, China; 2 Nankai University, Tianjin, China

## Abstract

With the rapid socioeconomic development and the increasing average life expectancy, China has shifted into an era of longevity. One significant feature of this era is the increasing size of Chinese centenarians and their health status are attracting increasing attention from academic communities. By using Chinese Longitudinal Healthy Longevity Survey (CLHLS), this paper employs Cox model to pinpoint the key influencing factors of mortality risk of Chinese centenarians. Our findings turn out that compared with the other elderly population, the mortality-risk determinants among centenarians are unique. The most important risk of the latter comes from their objective health status. The worse the health situation, the higher mortality risk is. Meanwhile, this rate is less affected by social and economic conditions. However, gender and smoking habit all play parts in the mortality risk of centenarians. This study comprehensively understands the key factors of the mortality risk of Chinese centenarians, which is of great significance for reducing centenarians’ mortality risks and enhancing their health and well-beings.

